# Supplementary material for: Comparative Effects of Efavirenz and Dolutegravir on Metabolomic and Inflammatory Profiles, and Platelet Activation of People Living with HIV: A Pilot Study
Source: Viruses. 2024 Sep 14;16(9):1462. doi: 10.3390/v16091462 (PMC11437493; doi:10.3390/v16091462)
Supplement: Supplementary file 1 [file viruses-16-01462-s001.zip › viruses-3186901-supplementary.pdf]

Table S1: Summary of the metabolites of interest of virally suppressed people living with HIV versus control individuals, irrespective of C-reactive protein concentration compared to those with C-reactive protein levels below 5 mg/L.

Metabolite	Efavirenz versus Control				Dolutegravir versus Control				Efavirenz versus Dolutegravir (paired)			
	Normal and elevated CRP		CRP below 5 mg/L		Normal and elevated CRP		CRP below 5 mg/L		Normal and elevated CRP		CRP below 5 mg/L	
	p-value	Fold Change	*p*-value	Fold Change	*p*-value	Fold Change	*p*-value	Fold Change	*p*-value	Fold Change	*p*-value	Fold Change
Acetoacetic Acid	**0.0043**	**0.0321**	**0.0193**	**0.0987**	0.1047	0.2045	0.4008	0.5301	-	-	-	-
Acetone	**0.0030**	**0.0304**	0.1012	0.2304	0.2948	0.4030	0.1196	0.3197	-	-	-	-
AMP	**0.0231**	**0.0631**	**0.0089**	**0.0609**	0.5315	0.6226	0.4008	0.5301	-	-	-	-
Creatinine	**<0.0001**	**0.0029**	**0.0036**	**0.0551**	**0.0060**	**0.0409**	**0.0303**	0.1772	-	-	-	-
1,7-Dimethylxanthine	0.3648	0.4674	**0.0243**	0.1105	**0.0071**	**0.0413**	0.1404	0.3197	-	-	-	-
3-Hydroxybutyric Acid	0.6601	0.7122	0.2488	0.3518	0.8352	0.9011	0.8201	0.8406	0.49609	0.8063	**0.0625**	**0.6406**
Formic Acid	**0.0097**	**0.0397**	**0.0007**	**0.0292**	**<0.0001**	**0.0004**	**0.0017**	**0.0353**	-	-	-	-
Glucose	**0.0060**	**0.0321**	**0.0706**	**0.1810**	**0.0341**	**0.0874**	0.1012	0.3197	-	-	-	-
Glycolic Acid	**0.0011**	**0.0228**	**0.0067**	**0.0551**	**<0.0001**	**0.0020**	**0.0067**	**0.0918**	-	-	-	-
Glycine	**0.0341**	**0.0699**	0.2488	0.3518	0.1531	0.2324	0.8796	0.8796	-	-	-	-
Lactic Acid	**0.0231**	**0.0631**	**0.0067**	**0.0551**	0.7637	0.1171	0.1893	0.3375	**0.0547**	**0.7550**	0.3125	0.8542
Leucine	**0.0300**	**0.0684**	0.1893	0.3235	**0.0131**	**0.0538**	0.1196	0.3197	-	-	-	-
Lysine	**0.0071**	**0.0321**	**0.0151**	**0.0884**	**0.0175**	**0.0635**	0.1893	0.3375	-	-	-	-
Myo-inositol	**0.0020**	**0.0278**	**0.0943**	**0.2417**	0.1272	0.2267	0.5426	0.6357	**0.0117**	**0.4805**	0.1875	0.7688
Urea	**0.0131**	**0.0414**	**0.0049**	**0.0551**	**0.0030**	**0.0304**	**0.0017**	**0.0353**	-	-	-	-
Valine	**0.0113**	**0.0414**	0.2488	0.3518	**0.0097**	**0.0496**	**0.0667**	**0.3223**	-	-	-	-

Abbreviations: AMP: Adenosine monophosphate; CRP: C-reactive protein.

Values in bold denote significance.

Table S2: Summary of cytokines, chemokines, and growth factors of virally suppressed people living with HIV versus control individuals, irrespective of C-reactive protein concentration compared to those with C-reactive protein levels below 5 mg/L.

	**Efavirenz versus Control**		**Dolutegravir versus Control**		**Efavirenz versus Dolutegravir (paired)**	
**Marker**	**Normal and elevated CRP**	**CRP below 5 mg/L**	**Normal and elevated CRP**	**CRP below 5 mg/L**	**Normal and elevated CRP**	**CRP below 5 mg/L**
IL-1β	0.6529	0.8434	0.5509	0.2750	0.8885	0.1250
IL-4	0.4218	0.2162	0.3694	0.2538	>0.9999	0.9999
IL-6	0.2081	0.1963	0.8126	0.9999	**0.0853**	**0.0625**
IL-8	0.5872	0.1512	0.4492	0.2750	0.7263	0.2012
IL-9	0.4649	0.6331	0.5244	0.8052	0.9102	0.4375
IL-10	0.5047	0.1358	0.4885	0.5830	0.8203	0.6250
IL-12	0.5384	0.1519	0.6528	0.3470	0.5541	0.2012
IL-13	0.6877	0.8048	0.3555	0.3473	0.9326	0.1003
IL-17	0.6703	0.4004	0.4780	0.7665	0.9442	0.6250
IL-1Rα	0.3445	0.3228	0.2469	0.7666	0.9527	0.3430
Eotaxin	0.7637	0.7028	0.9812	0.8436	0.3008	0.1875
FGF Basic	0.2172	0.1812	0.1612	0.3982	0.5703	0.8125
G-CSF	0.6706	**0.0933**	**0.0983**	**0.0604**	0.2340	0.9999
GM-CSF	0.2833	**0.0669**	0.2742	0.6911	0.4065	**0.0625**
IFN-γ	0.4040	0.1947	0.2968	0.1645	0.9102	0.6250
TNF-α	0.3571	0.1521	0.1784	0.4011	0.8203	0.6250
IP-10	0.1826	0.2887	0.4942	0.5028	0.8203	0.9999
MCP-1	0.2674	0.5871	0.4788	0.4006	0.5703	0.8125
MIP-1α	0.4072	0.1246	0.1302	**0.0598**	0.2936	0.9999
MIP-1β	0.7235	0.7750	0.5092	0.9241	0.2500	0.8125
PDGF-BB	**0.0426**	**0.0842**	0.3574	0.8049	0.6523	0.4375
sCD40L	**0.0077**	**0.0383**	**0.0659**	0.8436	0.8203	0.1875
RANTES	**0.0077**	**0.0383**	**0.0659**	0.8436	0.8203	0.1875

Abbreviations: α: alpha; β: beta; CRP: C-reactive protein; FGF: fibroblast growth factor; IFN: interferon; IL: interleukin; IP: interferon gamma-induced protein; MIP: macrophage inflammatory protein; γ: gamma; G-CSF: granulocyte colony-stimulating factor; GM-CSF: granulocyte-monocyte colony-stimulating factor; MCP: monocyte chemoattractant protein; PDGF: platelet-derived growth factor; RANTES: regulated on activation, normal T-cell expressed and secreted; sCD40L: soluble cluster of differentiation 40 ligand, TNF: tumor necrosis factor.

Values in bold denote significance.

Table S3: The median and interquartile ranges of the investigated cytokines and growth factors of virally suppressed people living with HIV versus controls irrespective of C-reactive protein concentration and those with C-reactive protein levels below 5 mg/L.

	Efavirenz		Dolutegravir		Control	
**Marker**	**CRP below and above 5 mg/L**	**CRP below 5 mg/L**	**CRP below and above 5 mg/L**	**CRP below 5 mg/L**	**CRP below and above 5 mg/L**	**CRP below 5 mg/L**
IL-1β	1.07 (0.68 – 1.46)	1.36 (0.98 – 2.71)	1.07 (0.98 – 1.07)	1.07 (0.78 – 1.07)	1.37 (0.52 – 1.63)	1.37 (0.47 – 1.63)
IL-4	2.58 (1.95 – 3.35)	2.91 (2.33 – 3.35)	2.69 (2.07 – 3.44)	2.69 (2.35 – 3.44)	2.42 (1.72 – 2.97)	2.42 (1.63 – 2.77)
IL-6	1.91 (1.36 – 2.10)	1.91 (1.00 – 2.10)	1.54 (1.17 – 1.73)	1.17 (0.82 – 1.64)	1.45 (0.99 – 1.91)	1.36 (0.99 – 1.97)
IL-8	5.20 (3.61 – 8.61)	8.04 (5.62 – 9.73)	4.63 (4.34 – 7.89)	5.20 (4.63 – 7.89)	4.63 (2.28 – 8.96)	4.63 (1.83 – 8.75)
IL-9	145.52 (127.97 – 161.00)	159.81 (117.45 – 165.26)	149.82 (118.45 – 153.63)	132.56 (118.45 – 153.15)	144.08 (109.48 – 149.70)	144.08 (109.48 – 151.49)
IL-10	1.79 (0.97 – 4.83)	3.42 (1.38 – 6.45)	2.61 (0.96 – 3.83)	2.61 (0.96 – 3.42)	1.38 (0.13 – 3.93)	1.38 (0.13 – 3.93)
IL-12	5.06 (3.78 – 9.59)	7.09 (4.29 – 12.08)	5.06 (4.30 – 6.08)	5.06 (4.81 – 5.82)	4.81 (3.00 – 6.08)	4.04 (2.22 – 6.08)
IL-13	2.16 (1.35 – 2.61)	2.60 (1.82 – 4.75)	1.93 (1.70 – 2.38)	1.70 (1.59 – 2.50)	2.27 (1.59 – 2.91)	2.27 (1.23 – 2.91)
IL-17	4.60 (4.20 – 8.36)	6.99 (3.79 – 8.55)	5.80 (4.20 – 8.16)	5.00 (3.79 – 6.98)	5.00 (3.38 – 7.18)	4.80 (2.77 – 7.18)
IL-1Rα	427.40 (370.22 – 480.06)	427.40 (370.22 – 509.86)	390.01 (340.05 – 505.39)	350.43 (318.88 – 426.51)	350.43 (223.74 – 488.59)	308.10 (210.94 – 458.61)
Eotaxin	32.25 (23.60 – 41.66)	32.25 (24.44 – 40.09)	33.63 (22.10 – 51.61)	33.63 (27.64 – 51.61)	37.55 (19.69 – 46.76)	35.91 (19.57 – 46.76)
FGF Basic	22.74 (22.09 – 29.14)	27.52 (22.72 – 32.28)	25.21 (24.61 – 28.62)	25.21 (24.61 – 27.46)	20.76 (18.65 – 29.71)	22.09 (18.65 – 29.71)
G-CSF	64.69 (48.58 – 71.48)	68.10 (59.42 – 72.32)	71.48 (56.82 – 82.96)	71.48 (55.93 – 82.96)	52.33 (41.56 – 77.29)	48.66 (39.62 – 73.98)
GM-CSF	1.68 (0.95 – 2.13)	2.04 (1.59 – 2.65)	1.41 (1.19 – 1.68)	1.32 (1.09 – 1.77)	1.05 (0.95 – 1.75)	1.23 (0.88 – 1.75)
IFN-γ	11.04 (9.69 – 12.87)	12.37 (10.03 – 14.50)	11.37 (10.37 – 12.37)	11.71 (10.71 – 12.37)	9.69 (7.61 – 13.20)	9.69 (6.91 – 12.45)
TNF-α	47.82 (44.60 – 54.96)	54.17 (47.02 – 57.70)	51.01 (45.00 – 58.48)	47.82 (45.01 – 55.72)	46.21 (37.22 – 52.60)	42.97 (36.39 – 52.60)
IP-10	234.40 (183.91 – 252.98)	234.40 (153.15 – 258.21)	198.00 (164.58 – 227.22)	183 (154.68 – 330.35)	167.57 (119.92 – 242.74)	167.57 (115.34 – 242.74)
MCP-1	12.94 (10.19 – 17.19)	12.94 (8.71 – 17.19)	11.13 (9.47 – 17.71)	11.59 (9.465 – 17.71)	10.19 (8.18 – 16.48)	10.19 (8.74 – 18.32)
MIP-1α	1.51 (1.20 – 1.90)	1.78 (1.36 – 2.19)	1.71 (1.58 – 1.90)	1.90 (1.68 – 1.96)	1.28 (1.02 – 2.16)	1.28 (1.02 – 1.84)
MIP-1β	135.57 (129.53 – 138.50)	135.57 (112.77 – 138.12)	121.15 (102.46 – 137.56)	113.19 (94.56 – 149.32)	133.18 (109.25 – 138.69)	133.18 (107.82 – 138.26)
PDGF-BB	113.11 (90.29 – 155.44)	113.11 (104.88 – 153.61)	76.63 (44.15 – 338.02)	54.91 (17.65 – 153.28)	59.43 (28.50 – 116.78)	53.115 (28.50 – 91.67)
sCD40L	21.70 (19.74 – 25.90)	25.75 (19.85 – 55.18)	24.45 (17.56 – 54.71)	18.00 (16.38 – 25.83)	18.58 (16.51 – 19.69)	18.21 (15.86 – 19.53)
RANTES	434.00 (394.80 – 517.90)	515.00 (396.90 – 1103.60)	489 (351.20 – 1094.10)	360.00 (327.60 – 516.60)	371.60 (330.25 – 393.80)	364.20 (317.20 – 390.60)

Abbreviations: α: alpha; β: beta; CRP: C-reactive protein; FGF: fibroblast growth factor; IFN: interferon; IL: interleukin; IP: interferon gamma-induced protein; MIP: macrophage inflammatory protein; γ: gamma; G-CSF: granulocyte colony-stimulating factor; GM-CSF: granulocyte-monocyte colony-stimulating factor; MCP: monocyte chemoattractant protein; PDGF: platelet-derived growth factor; RANTES: regulated on activation, normal T-cell expressed and secreted; sCD40L: soluble cluster of differentiation 40 ligand, TNF: tumor necrosis factor.
